# Prognostic Nomograms Predicting Survival in Patients With Locally Advanced Cervical Squamous Cell Carcinoma: The First Nomogram Compared With Revised FIGO 2018 Staging System

**DOI:** 10.3389/fonc.2020.591700

**Published:** 2020-10-20

**Authors:** Xi Yang, Jusheng An, Yuanyuan Zhang, Yong Yang, Siye Chen, Manni Huang, Lingying Wu

**Affiliations:** ^1^Department of Gynecological Oncology, National Cancer Center/National Clinical Research Center for Cancer/Cancer Hospital, Chinese Academy of Medical Sciences and Peking Union Medical College, Beijing, China; ^2^Department of Radiation Oncology, National Cancer Center/National Clinical Research Center for Cancer/Cancer Hospital, Chinese Academy of Medical Sciences and Peking Union Medical College, Beijing, China

**Keywords:** cervical squamous cell carcinoma, locally advanced stage, FIGO 2018 stage, nomogram, prognostic model

## Abstract

**Objectives:**

To develop nomograms to assess prognostic factors for 5-year overall survival (OS) and 5-year progression-free survival (PFS) in locally advanced cervical squamous cell carcinoma (LACSC).

**Methods:**

Overall, 618 patients with LACSC were included in this retrospective analysis. Nomograms for 5-year OS and PFS were developed based on Cox proportional hazards regression models. Concordance index (C-index) and calibration curves were used to define the predictive and discriminatory capacity of the nomogram. A comparison between the nomogram and the International Federation of Gynecology and Obstetrics (FIGO) staging system was conducted using time-dependent receiver operating characteristic (tROC) and area under the curve (tAUC).

**Results:**

Multivariate analysis identified several prognostic factors for OS including squamous cell carcinoma antigen (SCC-Ag), body mass index (BMI), tumor size, pelvic wall involvement, and para-aortic lymph node metastasis (PALNM). Prognostic factors for PFS included BMI, hemoglobin (HGB), tumor size, pelvic wall involvement, pelvic lymph node metastasis (PLNM) and PALNM. Following bootstrap correction, the C-index of OS and PFS was 0.713 and 0.686, respectively. These nomograms showed superior performance compared with the FIGO 2009 and 2018 staging schema.

**Conclusions:**

Nomograms were developed to identify prognostic factors for 5-year OS and PFS in patients with LACSC. These nomograms showed good prognostication and are more comprehensive in predicting survival outcomes than existing staging criteria.

## Introduction

Cervical cancer is the fourth most common cancer in females, with around 570,000 new cases of cervical cancer diagnosed per year worldwide, leading to ~300,000 deaths every year (1–3). In 2015, a total of 98.9 thousand new cases and 30.5 thousand deaths from cervical cancer were reported in China (4). According to existing evidence, concurrent chemoradiotherapy (CCRT) is the standard treatment for patients with locally advanced cervical cancer (LACC). Meanwhile, multiple clinical trials of investigational therapies have been conducted. The inclusion criteria of most clinical trials are based on the staging system of the International Federation of Gynecology and Obstetrics (FIGO). However, the FIGO 2009 system is gynecologic examination-based and does not cover lymph node status. Furthermore, with a concordance index (C-index) < 0.6, it is not accurate enough for predicting survival (5, 6).

The staging guidelines for cervical cancer have gradually shifted over the past twenty years (7, 8). In 2018, FIGO released a new staging system, which for the first time allowed the use of imaging modalities and pathologic assessment for staging (1). The most significant change is the definition of nodal status. Patients with metastasis to pelvic lymph nodes are defined as stage IIIC1, and para-aortic lymph node involvement is categorized as stage IIIC2. Lymph node metastasis is a crucial prognostic factor related to decreased survival in patients with LACC (9, 10). This new staging system clearly reflects the importance of lymph node metastasis in cervical cancer (11).

The modified FIGO 2018 staging system is still under evaluation especially with regards to the definitions of stage IIIC disease. There have been several studies to validate the prognostic performance of this revised staging system; however, patients with stage IIIC1 disease present with heterogeneous characteristics and varied survival rates (12). In addition, superior survival was reported in patients with stage IIIC1 compared with stage IIIA-B disease (13). These findings imply that multiple factors in addition to nodal status play crucial roles in survival.

In light of the new classification, we performed a retrospective study of patients with LACC who were treated with radiotherapy at our institution from 2010 to 2013. The primary objective of our study was to establish comprehensive prognostic nomograms and compare them with the FIGO 2018 cervical cancer staging system. We constructed nomograms with a special focus on squamous cell carcinoma, which is the major pathological subtype of cervical cancer.

## Methods

### Case Selection

Patients with histologically conﬁrmed, FIGO 2009 stage IB–IVA cervical squamous cell carcinoma who underwent radiotherapy at our institute from January 2010 to December 2013 were eligible. All other pathological types were excluded. Patients with secondary tumors were also excluded. Additional exclusion criteria included incomplete treatment or incomplete medical records. The tumor stage was determined by both pelvic examination and magnetic resonance imaging (MRI). Lymph node status and distant metastases were assessed by computed tomography (CT). The pretreatment evaluation included age, body mass index (BMI), pretreatment hemoglobin (HGB), level of squamous cell carcinoma antigen (SCC-Ag), node status, tumor size, and pelvic wall involvement by physical examination. Related clinical characteristics, including original FIGO 2009 status, were recorded. All patients were reassessed retrospectively according to the new FIGO 2018 staging system, based on MRI and CT reports. Lymph node metastasis was recorded if the lymph node measured ≥1.0 cm in short axis (14). Other features such as shape and the absence of the fatty hila were considered by radiologists. The study was approved by the Ethics Board of the Cancer Hospital, Chinese Academy of Medical Sciences (CAMS).

### Treatment

The standard treatment for LACC is CCRT, while radiotherapy alone is indicated in older patients or those with insufficient renal function. Neoadjuvant chemotherapy was prescribed for specific patients with smaller tumor size when surgery opportunity was considered. Adjuvant chemotherapy was prescribed if considered necessary by clinicians based on the disease situation, such as a low grade of pathological differentiation, inadequate tumor shrinkage or abnormal SCC-Ag after radiotherapy. The standard chemotherapy regimen was paclitaxel combined with cisplatin or other platinum-based therapies.

Radiotherapy consisted of whole pelvic external beam radiation therapy (EBRT) and high-dose-rate brachytherapy (HDR-BT). A total EBRT dose of 45–50 Gy was delivered. The region of gross disease, the parametrial, uterosacral ligaments and pelvic nodal region at risk was covered by the clinical target volume (CTV) of EBRT. An adequate margin (3–5 mm) was applied to create the planning target volume (PTV) avoiding organs at risk (OAR). Inguinal nodes were also included when the lower third of the vagina was involved. Common iliac region was covered in patients with pelvic lymph node involvement. If metastasis of common iliac or para-aortic nodes were detected, extended-field radiotherapy was delivered. Enlarged lymph nodes received an extra boost irradiation dose of 10–15 Gy. The OAR delineation and planning constraints were as follows: rectum: maximal dose <52 Gy, volume receiving >50 Gy (V50) <20%; sigmoid: maximal dose <52 Gy, V40 <60%; bladder: V50 <20%; intestines: maximal dose <52 Gy, V40 <50%; spinal cord: maximal dose <40 Gy; femoral heads: V30 <30%, pelvic bone marrow: V30 <50%; kidneys:V20 <20%. High-dose-rate brachytherapy was performed weekly (5–7 Gy each). A cumulative dose to point A was 80–85 Gy (equivalent dose in 2 Gy/f, EQD2) according to disease stage. Platinum-containing chemotherapy during EBRT was the recommended concurrent chemotherapy regimen. More information on the details of radiotherapy can be found in the article previously published by our institution (15).

### Statistical Analysis

As the endpoints of this study, overall survival (OS) was defined as the time from the beginning of treatment to the date of death by any cause or the most recent follow-up. Progression-free survival (PFS) was defined as the time from the start of treatment to disease progression, relapse, or death.

The Kaplan-Meier method was used to generate PFS and OS curves. The subgroups of PFS and OS were compared by the Kaplan-Meier method and the log-rank test, and P < 0.05 was considered statistically significant. A Cox proportional hazards regression model was used to adjust for potential confounding factors such as age, BMI, HGB, SCC-Ag, tumor size by physical examination, pelvic wall involvement, and nodal status. Age, BMI, HGB, SCC-Ag, and tumor size were both modeled as continuous and categorical variables, and other variables were modeled as categorical variables. Hazard ratios (HRs) and 95% confidence intervals (CIs) for these variables were calculated. The results of multivariate analysis were used to establish nomograms for 5-year OS and PFS. Independent significant variables were used to develop the nomograms. The effect of selected variables on survival probability was visualized, and internal validation was performed using the bootstrap method. C-index and calibration curves were used to define predictive and discriminatory capacity. The time-dependent receiver operating characteristics (tROC) and corresponding area under the curve (tAUC) were used to compare the prognostic accuracy of the nomograms and the FIGO staging system. The tAUC of ROC was calculated at 12-month intervals from the 12^th^ to the 72^nd^ month for both nomograms and the two FIGO staging systems. Inverse probability of censoring weighting estimation was used to calculate tAUC. Statistical analyses were performed using SPSS Statistics, Version 25.0 and R software, version 3.4.4. Packages for “survival”, “rms”, and “timeROC” were used (http://www.r-project.org/).

## Results

A total of 613 patients were identified who met the inclusion criteria. The characteristics of all enrolled patients are summarized in **Table 1**. The median age of patients was 54 years (range: 30–89 years). Low BMI (<18.5 kg/m^2^) was observed in 23 patients (8.8%), while 417 patients had low HGB (68%). Pelvic lymph nodes were involved in 52.2% of patients, and para-aortic lymph node metastasis (PALNM) was observed in 5.7% of patients. Pelvic wall involvement was present in 39.2% of patients. The majority of patients had stage IIB and IIIB disease according to the FIGO 2009 staging system (n = 324, 52.8% and n = 232, 37.8%, respectively), or IIB and IIIC1r disease based on FIGO 2018 criteria (n = 193, 31.5% and n = 282, 46%, respectively). All patients received complete radiotherapy, of which 317 patients (51.7%) received traditional two-dimension radiotherapy and 296 patients (48.3%) received intensity modulated radiotherapy (IMRT). Overall, 110 patients (17.9%) received 1–3 cycles of neoadjuvant chemotherapy. Most patients received concurrent chemotherapy during radiotherapy (n = 547, 89.2%). Over half of the patients were treated with concurrent weekly cisplatin monotherapy (n = 324, 52.8%). Alternative regimens included cisplatin plus 5-fluorouracil (n = 23, 3.8%) or paclitaxel plus cisplatin (n = 200, 32.6%). Ninety-three patients (15.2%) received 1–3 cycles of adjuvant chemotherapy after radiotherapy.

**Table 1:** Clinical characteristics of patients and distribution of FIGO staging system.

Characteristic	No. Patients	%
Age (year)		
31–40	32	5.2
41–50	172	28.1
51–60	243	39.6
61–70	134	21.9
≥71	32	5.2
BMI (kg/m^2^)		
<18.5	23	3.8
18.5~23.9	285	46.5
24~29.9	274	44.7
≥30	31	5.1
SCC-Ag (ng/ml)		
≤1.5	108	17.6
1.6~5.0	179	29.2
5.1~10.0	113	18.4
10.1~20	93	15.2
>20	120	19.6
HGB (g/L)		
<60	15	2.4
60-89	135	22.0
90–109	267	43.6
≥110	196	32.0
Tumor size (cm)		
≤4	235	38.3
4.1~5.9	276	45.1
≥6	102	16.6
PLNM		
No	293	47.8
Yes	320	52.2
PALNM		
No	578	94.3
Yes	35	5.7
Pelvic wall involvement		
No	373	60.8
Yes	240	39.2
FIGO 2009 stage		
IB	26	4.3
IIA	17	2.7
IIB	324	52.9
IIIA	6	1.0
IIIB	232	37.8
IVA	8	1.3
FIGO 2018 stage		
IB	16	2.6
IIA	5	0.8
IIB	193	31.5
IIIA	3	0.5
IIIB	71	11.6
IIIC1r	282	46.0
IIIC2r	35	5.7
IVA	8	1.3
Radiotherapy		
2D	317	51.7
IMRT	296	48.3
Neoadjuvant chemotherapy		
No	503	82.1
Yes	110	17.9
Concurrent chemotherapy		
No	66	10.8
Yes	547	89.2
Platinum	324	52.8
Paclitaxel + platinum	200	32.6
Platinum + 5FU	23	3.8
Adjuvant chemotherapy		
No	520	84.8
Yes	93	15.2

BMI, body mass index; SCC-Ag, squamous cell carcinoma antigen; HGB, hemoglobin; PLNM, pelvic lymph node metastasis; PALNM, para-aortic lymph node metastasis; FIGO, International Federation of Gynecology and Obstetrics; 2D, two dimensions radiotherapy; IMRT, intensity modulated radiotherapy; 5FU, 5-fluorouracil.

**Figure 1:**
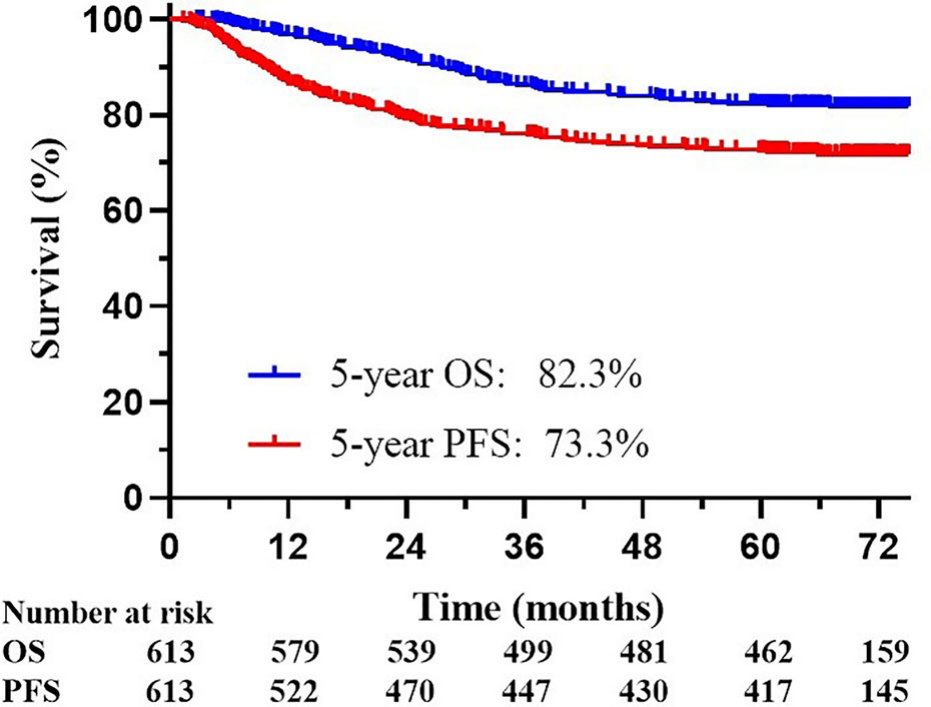
Kaplan-Meier overall survival (OS) curve and progression-free survival (PFS) curve for all 613 patients included in the analyses.

The median follow-up time was 61.5 months. The OS and PFS in the follow-up period was 82.3% and 73.3%, respectively (**Figure 1**). Survival information according to FIGO 2009 and 2018 staging are displayed in **Table 2**. During follow-up, 113 patients experienced disease progression, including 88 distant metastases (13 patients had both local-regional and distant recurrence), 12 regional recurrences (3 patients experienced both regional and local recurrence), and 13 local recurrences.

**Table 2:** Overall survival and progression-free survival according to FIGO staging system.

	FIGO2009			FIGO2018		
	n	5y-OS (%)	5y-PFS (%)	n	5y-OS (%)	5y-PFS (%)
IB	26	88.3	84.6	16	80.8	75.0
IIA	17	88.2	82.4	5	60.0	60.0
IIB	324	89.7	81.1	193	93.5	87.1
IIIA	6	83.3	66.7	3	100	100.0
IIIB	232	71.9	59.2	71	72.2	65.2
IIIC1r				282	82.5	70.3
IIIC2r				35	50.3	27.8
IVA	8	53.6	60.0	8	53.6	60.0

**Figure 2:**
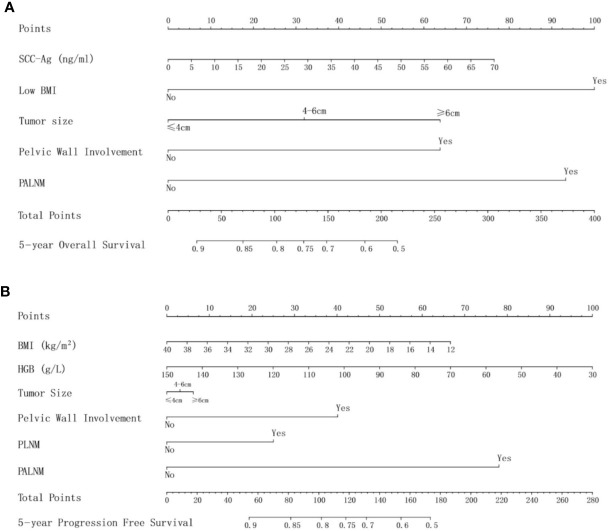
**(A)** nomogram for 5-year overall survival; **(B)** nomogram for 5-year progression-free survival. Abbreviations: SCC-Ag, squamous cell carcinoma antigen; BMI, body mass index; HGB, hemoglobin; PLNM, pelvic lymph node metastasis; PALNM, para-aortic lymph node metastasis.

**Figure 3:**
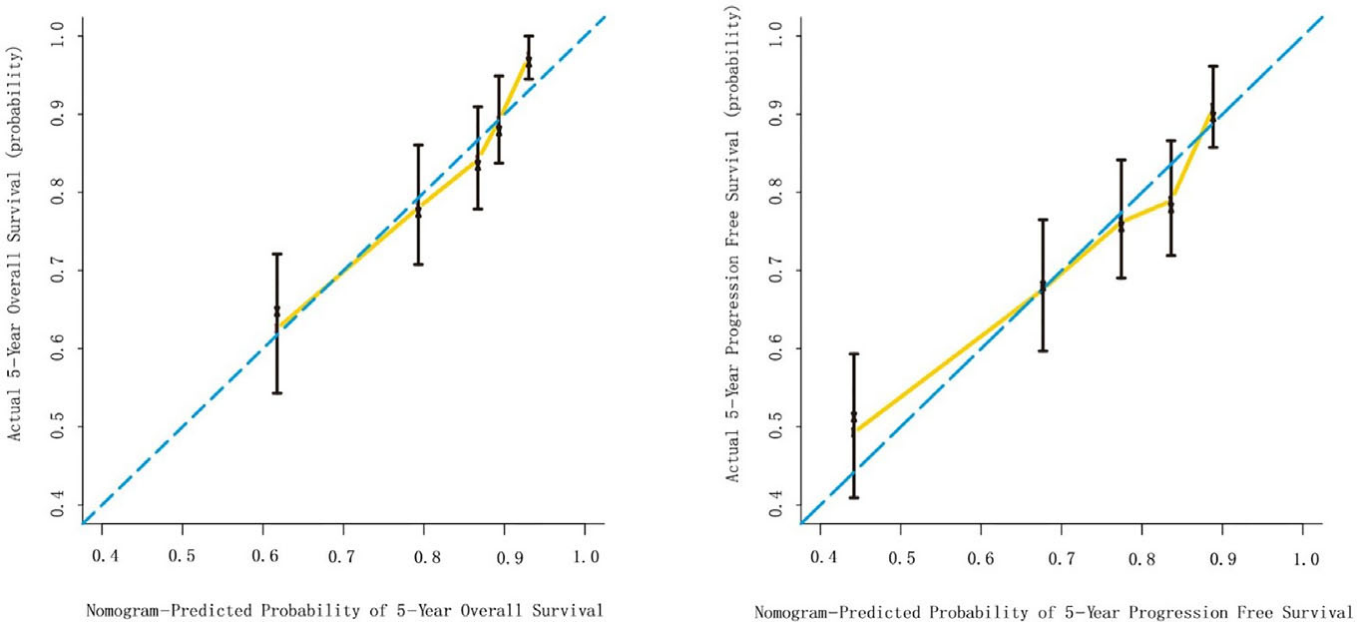
Internal validation of the nomogram to predict overall survival (OS) and progression-free survival (PFS) likelihoods in patients with LACSC. The nomogram-predicted probability of OS and PFS is plotted on the x axis; the actual OS and PFS is plotted on the y axis. **(A)** calibration curve for 5-year OS; **(B)** calibration curve for 5-year PFS.

**Figure 4:**
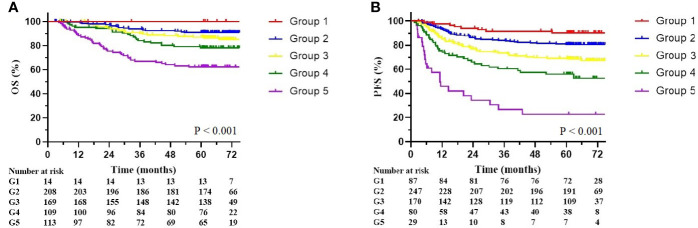
Overall survival and progression-free survival stratified by nomogram score. **(A)** Kaplan-Meier overall survival curves. **(B)** Kaplan-Meier progression-free survival curves. The groups are divided by nomogram score (Group 1: less than 50; Group 2: 50–100; Group 3: 100–150; Group 4: 150–200; Group 5: over 200).

**Figure 5:**
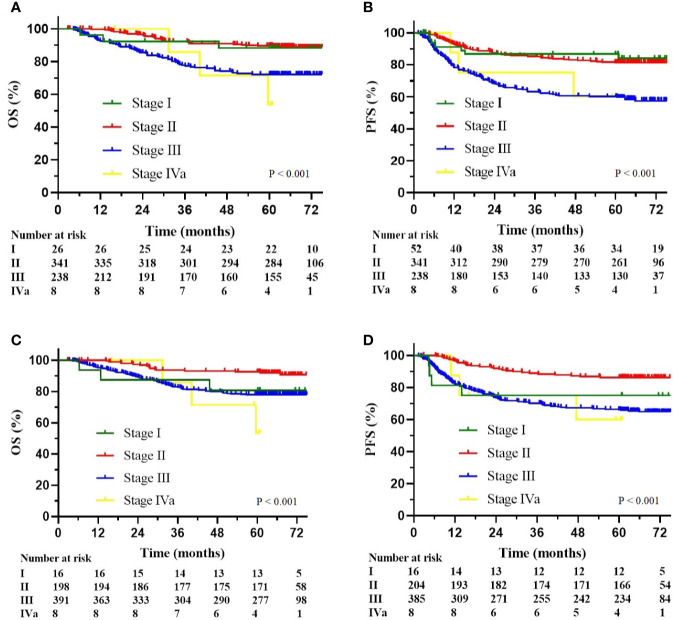
**(A)** Kaplan-Meier overall survival curves according to the FIGO 2009 stage; **(B)** Kaplan-Meier progression-free survival curves according to the FIGO 2009 stage; **(C)** Kaplan-Meier overall survival curves according to the FIGO 2018 stage; **(D)** Kaplan-Meier progression-free survival curves according to the FIGO 2018 stage.

**Figure 6:**
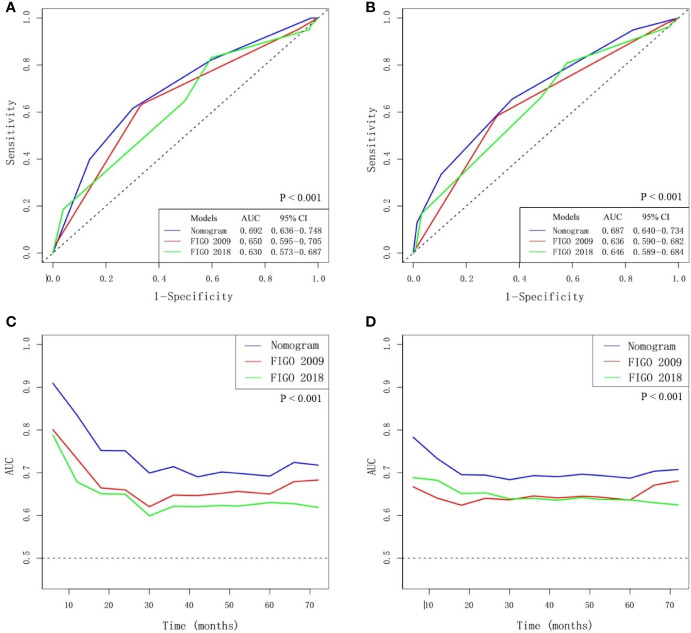
Comparison of nomogram and FIGO staging system. **(A)** AUC for predicting 5-year overall survival (OS). **(B)** AUC for predicting 5-year progression-free survival (PFS). **(C)** The time-dependent AUC between 6 and 72 months for OS. **(D)** The time-dependent AUC between 6 and 72 months for PFS.

The multivariate Cox analysis showed that SCC-Ag, low BMI, tumor size, pelvic wall involvement, and PALNM were significantly correlated with OS (**Table 3**). Furthermore, BMI, HGB, tumor size, pelvic wall involvement, PLNM, and PALNM were correlated with PFS (**Table 4**). Based on this, independent prognostic factors were used to establish the 5-year OS and PFS nomograms (**Figure 2**). The C-index of the OS nomogram was 0.713, and the concordance index of the PFS nomogram was 0.686 (**Figure 3**). Acceptable agreement between the predicted and actual probability of 5-year OS and PFS was observed in calibration plots. Patients were subdivided into five groups according to the nomogram scores (**Table 5**) and a survival curve was drawn (all, P < 0.001; **Figure 4**). The discrimination of both FIGO 2009 and 2018 staging system for OS and PFS was not visually ideal (all, P < 0.001; **Figure 5**). The 5-year OS for stage IB, IIA, IIB, and IIIA disease according to FIGO 2009 were similar. The survival outcomes for each group according to FIGO 2018 staging were not always lower in patients with more advanced disease (**Table 2**). The C-index of the nomogram for OS was 0.713 (95% CI: 0.666–0.760), while the C-index for FIGO 2009 staging and FIGO 2018 staging was 0.636 (95% CI 0.587–0.685) and 0.616 (95% CI 0.565–0.667), respectively. Similarly, for PFS, the C-indices for FIGO 2009 staging (0.621, 95% CI 0.582–0.660) and FIGO 2018 staging (0.628, 95% CI 0.587–0.669) were lower than the nomogram (0.686, 95% CI 0.645–0.727). The AUC of the nomogram for predicting 5-year OS (0.692, 95% CI: 0.636–0.748) was signiﬁcantly superior compared with the FIGO 2009 staging system (0.650, 95% CI: 0.595–0.705; P = 0.037), and FIGO 2018 staging system (0.630, 95% CI: 0.573–0.687; P = 0.043) (**Figure 6A**). Similarly, the nomogram for PFS had a signiﬁcantly higher AUC (0.687, 95% CI: 0.640–0.734) compared with the FIGO 2009 staging system (0.636, 95% CI: 0.590–0.682; P = 0.002), and FIGO 2018 staging system (0.646, 95% CI: 0.589–0.684; P = 0.009) (**Figure 6B**). Additionally, the tAUC between 6 and 72 months was consistently superior compared with the FIGO system for both OS and PFS (all, P < 0.001; **Figures 6C, D**). The results suggest that these nomograms provide an accurate and ready-to-use risk model for stratifying and discriminating OS and PFS in patients with locally advanced cervical squamous cell carcinoma (LACSC).

**Table 3:** Multivariate analysis for overall survival.

Variable	Multivariate		
	HR	95%CI	P
SCC-Ag (ng/ml)	1.010	1.001–1.021	0.026
BMI (<18.5 kg/m^2^ vs. ≥18.5 kg/m^2^)	2.622	1.309–5.251	0.007
Tumor size (cm)			
≤4			
4.1~5.9	1.785	1.088–2.929	0.022
≥6	1.934	1.072–3.491	0.029
Pelvic wall involvement (yes vs. no)	1.907	1.261–2.883	0.002
PALNM (yes vs. no)	2.460	1.408–4.297	0.002

SCC-Ag, squamous cell carcinoma antigen; BMI, body mass index; PALNM, para-aortic lymph node metastasis; HR, hazard ratio; CI, confidence interval.

**Table 4:** Multivariate analysis for progression-free survival.

Variable	Multivariate		
	HR	95%CI	P
BMI (kg/m^2^)	0.965	0.924–1.007	0.098
HGB (g/L)	0.987	0.978–0.996	0.004
Tumor size (cm)			
≤4			
4.1~5.9	1.401	0.967–0.996	0.075
≥6	1.063	0.660–1.713	0.802
Pelvic wall involvement (yes vs. no)	1.905	1.365–2.660	0.002
PLNM (yes vs. no)	1.490	1.050–2.114	0.026
PALNM (yes vs. no)	3.266	2.047–5.208	<0.001

BMI, body mass index; HGB, hemoglobin; PLNM, pelvic lymph node metastasis; PALNM, para-aortic lymph node metastasis; HR, hazard ratio; CI, confidence interval.

**Table 5:** Overall survival and progression-free survival according to nomogram score groups.

	Nomogram			
	n	5y-OS(%)	n	5y-PFS(%)
Group 1 (0–50)	13	100	87	90.5
Group 2 (50–100)	208	90.9	247	80.6
Group 3 (100–150)	169	87.1	170	69.1
Group 4 (150–200)	109	78.0	80	55.9
Group 5 (>200)	113	62.2	29	22.9

## Discussion

Cervical cancer was the fourth most common cause of cancer mortality worldwide in 2015. At present, the FIGO system remains the predominate basis for staging of cervical cancer in clinical practice. Staging according to the old system (FIGO staging system 2009) was not accurate. Additionally, it did not include lymph node status, which is an important component for defining prognosis and optimal treatment. The new FIGO 2018 staging system highlights the importance of nodal involvement in cervical cancer patients (1). FIGO 2018 staging includes the presence of nodal disease (either radiological or pathological) in the classification, thus upstaging cervical cancer apparently confined to the uterine cervix from stage I–IIIB to stage IIIC.

According to validation by the Surveillance, Epidemiology, and End Results Program (SEER) database, survival of women with stage IIIC1 disease is superior to patients with stage IIIA or stage IIIB disease (12). Classification of all patients with positive lymph nodes into one category leads to a highly heterogeneous group, which highlights obvious limitations in the revised FIGO 2018 staging system, and a more comprehensive risk schema combining multiple factors may be necessary. Nomograms are widely used for risk assessment in many malignancies, including patients with LACC treated with definitive CCRT or radiotherapy (5, 6, 16). A nomogram is an easy-to-use model with the advantages of individual prediction and good visualization.

In this study, we established nomograms that were specifically designed for patients with squamous cell carcinoma. All pathological types except for squamous cell carcinoma were excluded to reduce the influence of confounding factors. The outcomes for patients with cervical squamous cell carcinoma receiving radiotherapy is thought to be better than those for patients with cervical adenocarcinoma or adenosquamous carcinoma, because these subtypes are more radioresistant (17, 18). This is one of the reasons that the 5-year OS in our study was relatively high. Patients who received neoadjuvant or adjuvant chemotherapy were not excluded from this study. CCRT remains the backbone of LACC treatment, and the role of neoadjuvant chemotherapy and adjuvant chemotherapy for specific patient groups remains under investigation (19–25). Although the treatment modality varied in our study, radiotherapy was the main therapeutic intervention.

Clinical variables related to tumor load, invasive potential, or treatment tolerance were evaluated, such as SCC-Ag, BMI, and HGB. Several studies have shown that SCC-Ag is of great significance in the prognosis of cervical squamous carcinoma (26–28). Similarly, in the present study, we observed a significant relationship between SCC-Ag and OS. As a continuous variable, the higher the level of SCC-Ag, the higher the nomogram score, which predicted a worse outcome.

There are also other prognostic factors which could be integrated into the current prognostic model. As the most frequent symptom of locally advanced cervical cancer, abnormal vaginal bleeding always leads to anemia. Anemia aggravates hypoxia inside the tumor, which has been shown to facilitate tumor proliferation and to enhance resistance to radiotherapy (29, 30). The first-line treatment for patients with LACC is CCRT. Low HGB was found to be a significant prognostic factor for PFS; however, low HGB was not related to OS in this study. We also showed that low BMI had an impact on clinical outcomes. Importantly, low BMI is in part caused by anemia which influences outcomes. Another hypothesis is that low BMI is related to tumor depletion. In this case, patients with larger tumor load have worse outcomes. We found that patients with low BMI had poor tolerance to treatment, which limits the efficacy of CCRT.

The extent of local tumor is an important factor for the survival of patients with cervical cancer (5, 10, 31–34). Local tumor extent in the FIGO staging system was subdivided into two parts as separate and independent existence, including tumor size by physical examination and pelvic wall involvement. Tumor size is not further divided in the FIGO system when it reaches >4 cm in diameter. Conversely, in this study, tumor size becomes significant when tumors > 4 cm and those > 6 cm in diameter are treated independently, which is consistent with previous research (31) that showed variation in survival outcomes between groups. Another important factor is pelvic wall involvement, which was classified as stage IIIB according to the FIGO 2009 staging system. Pelvic wall involvement was shown to be correlated with a poor disease-specific survival in previous studies (32, 35). In this study, the HR of pelvic wall involvement with regards to OS and PFS was 1.907 and 1.905, respectively, consistent with previous conclusions.

The presence of nodal metastases is highly prognostic for cervical cancer. PALNM is a known poor prognostic factor (36–38). Positive para-aortic nodal status is defined as IIIC2, which is an important clinical issue in new FIGO staging system. Similarly, in this study, PALNM proved to be significant for both OS and PFS with a high HR of 2.460 and 3.266, respectively. Both P values were extremely low (0.002 for OS and <0.001 for PFS). It is worth noting that, although PLNM was shown to be a prognostic factor in cervical cancer in previous studies (10, 32, 33, 39), PLNM in this study only correlates with an increased risk of progression, and does not impact OS. The reason was multicollinearity. This may be because PLNM is strongly associated with PALNM, SCC-Ag, tumor size or other elements. Otherwise, in this study, all LNM is recorded by imaging features. Enlarged lymph nodes noted by images sometimes are inflammatory which is commonly seen in patients with LACC. Survival in patients with stage IIIC1 disease also may differ according to different tumor load of involved lymph nodes, such as bulky lymphadenopathy versus microscopic metastasis. Thus, determination of lymph node status accurately is important.

We present the first nomogram to be compared with the modified 2018 FIGO staging system specifically for patients with LACSC. Compared with the FIGO system, our nomogram is more comprehensive with a high degree of concordance index. We included SCC-Ag, HGB, BMI, tumor extent, and nodal involvement without adding complexity for use.

By using systematic and effective methods, this risk model shows improved accuracy for prediction, which can facilitate better counseling and treatment for women with LACSC. However, as this was a single center and retrospective study, there is inevitable selection bias, as well as referral bias. Thus, external validation of the prognostic models is extremely important to test their prognostic signiﬁcance and identify potential weaknesses, which could be improved in subsequent studies. A lack of external validation is an important limitation of this study.

## Conclusion

In this study, nomograms were established for predicting 5-year OS and PFS in patients with LACSC. These nomograms had better calibration and discriminatory ability than both the FIGO 2009 and FIGO 2018 staging systems, and can be used for clinically meaningful prognostic assessment of patients with LACSC, although external validation is required.

## Data Availability Statement

The raw data supporting the conclusions of this article will be made available by the authors, without undue reservation.

## Ethics Statement

The studies involving human participants were reviewed and approved by Ethics Committee of National Cancer Center, Cancer Hospital, Chinese Academy of Medical Sciences and Peking Union Medical College. Written informed consent for participation was not required for this study in accordance with the national legislation and the institutional requirements.

## Author Contributions

All authors contributed to the article and approved the submitted version.

## Funding

This study was supported by the Beijing Hope Run Special Fund of Cancer Foundation of China (LC2018B04).

## Conflict of Interest

The authors declare that the research was conducted in the absence of any commercial or financial relationships that could be construed as a potential conflict of interest.
